# Novel Steroidal Glycosides from the Bulbs of *Lilium pumilum*

**DOI:** 10.3390/molecules200916255

**Published:** 2015-09-08

**Authors:** Yukiko Matsuo, Reina Takaku, Yoshihiro Mimaki

**Affiliations:** School of Pharmacy, Tokyo University of Pharmacy and Life Sciences, 1432-1 Horinouchi, Hachioji, Tokyo 192-0392, Japan; E-Mails: sept_couleur_r@yahoo.co.jp (R.T.); mimakiy@toyaku.ac.jp (Y.M.)

**Keywords:** *Lilium pumilum*, Liliaceae, steroidal glycosides

## Abstract

Examination of the bulbs of *Lilium pumilum* (Liliaceae) led to the isolation of four novel steroidal glycosides (**1**–**4**) with a 2,3,4-trisubstituted β-d-glucopyranosyl unit. In **1** and **3**, the α-l-arabinopyranosyl moiety is linked to C-3 of the inner trisubstituted β-d-glucopyranosyl group and is present as an usual ^4^C_1_ conformation. In contrast, in **2** and **4**, the α-l-arabinopyranosyl moiety, which is attached to C-4 of the inner trisubstituted β-d-glucopyranosyl group, is present as a ^1^C_4_ conformation. The structures of the new steroidal glycosides were determined based on the results of spectroscopic analyses, including two-dimensional (2D) NMR data and hydrolysis.

## 1. Introduction

*Lilium pumilum* D.C. (Liliaceae) is described in the Japanese Pharmacopoeia (16th edition) as a plant from which the crude drug Lilium Bulb is derived. Lilium Bulb has long been used as an antitussive and anti-inflammatory agent in traditional Chinese medicine [1]. The bulbs of *L. pumilum* (*L. tenuifolium* Fisch. ex Hook.) contain phenylpropanoid derivatives, such as regalosides A and D, and sterol glycosides, such as tenuifoliosides A and B [2]. Although plants belonging to the family Liliaceae are rich sources of bioactive steroidal glycosides, such as OSW-1 or galtonioside A with cytotoxic activities against tumor cells [3,4], few studies have focused on steroidal glycosides of *L. pumilum* [5]. Our preliminary analysis of the MeOH extract of *L. pumilum* bulbs suggests that it contains more steroidal glycosides. Here, we report four novel steroidal glycosides (**1**–**4**) with a trisubstituted β-d-glucopyranosyl unit isolated from the bulbs of *L. pumilum*. The structures of the new steroidal glycosides were determined based on the results of spectroscopic analysis, including two-dimensional NMR data, and hydrolysis followed by chromatographic and spectroscopic analyses.

## 2. Results and Discussion

**Figure 1:**
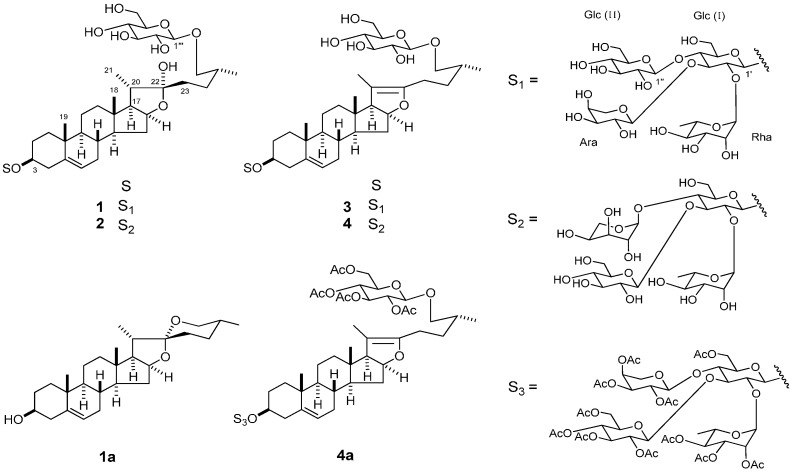
Steroidal glycosides from *Lilium pumilum*.

The bulbs of *L. pumilum* (1.4 kg fr. wt) were extracted with MeOH. The MeOH extract (65 g) was passed through a porous-polymer polystyrene resin (Diaion HP-20) column, and the MeOH-eluted fraction (1.8 g) was subjected to silica gel and octadecylsilanized (ODS) silica gel column chromatography (CC), giving **1**–**4** (Figure 1).

Compound **1** was obtained as an amorphous solid, and its molecular formula was assigned as C_56_H_92_O_27_ based on data from high-resolution electrospray ionization time-of-flight mass spectrometry (HR-ESI-TOF-MS) (*m*/*z* 1219.5674 [M + Na]^+^, calcd for 1219.5724) and ^13^C-NMR (56 carbon signals) spectra. The ^1^H-NMR spectrum of **1** showed two singlet signals for tertiary methyl groups at δ_H_ 1.04 and 0.89 (each s), two doublet signals for secondary methyl groups at δ_H_ 1.34 (d, *J* = 6.9 Hz) and 1.00 (d, *J* = 6.7 Hz), and five anomeric proton signals at δ_H_ 6.07 (br s), 5.56 (d, *J* = 5.4 Hz), 5.39 (d, *J* = 7.8 Hz), 4.92 (d, *J* = 7.6 Hz), and 4.82 (d, *J* = 7.8 Hz). The ^13^C-NMR spectrum showed a signal for a hemiacetal carbon at δ_C_ 110.6, signals for five anomeric carbon at δ_C_ 104.9, 103.2, 103.0, 102.4, and 99.8, and signals for four steroid methyl groups at δ_C_ 19.4, 17.4, 16.4, and 16.3 as shown in Table 1, which were characteristic of 22-hydroxyfurostanol glycosides [6]. Acid hydrolysis of **1** with 1 M HCl yielded **1a** as the aglycone and arabinose, glucose and rhamnose as the carbohydrate moieties. The monosaccharides and their absolute configurations were identified by direct HPLC analysis of the hydrolysate; l-arabinose, d-glucose and l-rhamnose, respectively. The aglycone (**1a**) was identified as (25*R*)-spirost-5-en-3β-ol (diosgenin) from its physical and spectroscopic data [7]. These NMR data, the chemical evidence, and the positive color reaction with Ehrlich’s reagent suggested that **1** was a 22-hydroxyprotodiosgenin bisdesmoside, the sugar moieties of which consisted of five monosaccharides. The ^1^H-^1^H COSY and 1D TOCSY spectra of **1** allowed the ^1^H-NMR chemical shifts, signal multiplet patterns, and coupling constants of the sugar moieties to be assigned as shown in Table 2. The ^1^H-NMR signals were associated with the corresponding one-bond coupled carbons using the HMQC and HSQC-TOCSY spectra, leading to the assignments of all the ^13^C-NMR chemical shifts of the sugar moieties. Comparing the ^13^C-NMR chemical shifts of each monosaccharide and reference methyl glycosides indicated the presence of a substituted β-d-glucopyranosyl (^4^C_1_) unit (Glc (I)), two β-d-glucopyranosyl (^4^C_1_) units (Glc (II) and Glc (III)), an α-l-arabinopyranosyl (^4^C_1_) unit (Ara), and an α-l-rhamnopyranosyl (^1^C_4_) unit (Rha) as the terminal glycosyl groups [8]. The ^13^C-NMR shifts of the inner Glc (I) moiety (δ_C_ 99.8, 78.9, 79.8, 72.5, 78.5, and 60.9) suggested that its C-2, C-3, and C-4 hydroxy groups were substituted with the other sugar moieties. The anomeric configurations of the Ara and Glc groups were ascertained as α and β, respectively, from the relatively large *J* values of their anomeric protons (5.4–7.8 Hz) [7]. For the Rha group, the ^13^C-NMR chemical shifts (δ_C_ 102.4, 72.4, 72.8, 73.8, 70.0, and 18.6) suggested an α-pyranoid anomeric form. The HMBC correlations between the anomeric proton (H-1) of Ara at δ_H_ 5.56 and C-3′ of Glc (I) at δ_C_ 79.8, between H-1′′ of Glc (II) at δ_H_ 5.39 and C-4′ of Glc (I) at δ_C_ 72.5, between H-1 of Rha at δ_H_ 6.07 and C-2′ of Glc (I) at δ_C_ 78.9, and between H-1′ of Glc (I) at δ_H_ 4.92 and C-3 of the aglycone at δ_C_ 77.9, indicated that the tribranched oligosaccharide of *O*-α-l-arabinopyranosyl-(1→3)-*O*-[β-d-glucopyranosyl-(1→4)]-*O*-[α-l-rhamnopyranosyl-(1→2)]-β-d-glucopyranosyl was linked to C-3 of the aglycone. A β-d-glucopyranosyl group (Glc (III)) linkage to the C-26 hydroxy group of the aglycone was confirmed by an HMBC correlation between H-1′′′ of Glc (III) at δ_H_ 4.82 (d, *J* = 7.8 Hz) and C-26 of the aglycone at δ_C_ 75.2. The NOE correlations between the signals of the H-20 proton at δ_H_ 2.21 and the H_2_-23 protons at δ_H_ 2.03 (2H) confirmed the C-22α configuration [9]. In the ^1^H-NMR spectrum, the difference chemical shifts of H_2_-26 geminal protons at δ_H_ 3.95 and δ_H_ 3.60, Δδ = 0.35 < 0.48 ppm, provided evidence for the (25*R*)-furostanol [10]. Thus, **1** was assigned as (25*R*)-26-[(β-d-glucopyranosyl)oxy]-22α-hydroxyfurost-5-*en*-3β-yl *O*-α-l-arabinopyranosyl-(1→3)-*O*-[β-d-glucopyranosyl-(1→4)]-*O*-[α-l-rhamnopyranosyl-(1→2)]-β-d-glucopyranoside.

**Table 1:** ^13^C-NMR spectral assignments for the aglycone moiety of **1**–**4** in C_5_D_5_N.

Position	1	2	3	4
1	37.5	37.1	37.5	37.6
2	29.9	29.9	29.9	29.9
3	77.9	78.0	77.8	77.9
4	39.7	39.7	38.6	38.6
5	140.8	140.8	140.8	140.7
6	121.8	121.8	121.8	121.6
7	32.4	32.4	32.4	32.2
8	31.6	31.6	31.4	31.5
9	50.3	50.3	50.3	50.2
10	37.1	37.1	37.1	37.1
11	21.0	21.1	21.2	21.2
12	39.9	39.9	39.6	39.7
13	40.7	40.7	43.4	43.4
14	56.5	56.5	54.9	54.9
15	32.1	32.2	34.4	34.5
16	81.3	81.2	84.5	84.4
17	63.8	64.1	64.5	64.5
18	16.4	16.4	14.1	14.3
19	19.4	19.3	19.4	19.5
20	40.6	40.6	103.6	103.6
21	16.3	16.4	11.7	11.7
22	110.6	110.6	152.3	152.4
23	37.1	37.0	23.6	23.8
24	28.3	28.3	31.4	31.5
25	34.2	34.2	33.5	33.6
26	75.2	75.2	75.2	75.3
27	17.4	17.4	17.3	17.3

**Table 2:** ^1^H- and ^13^C-NMR spectral assignments for the sugar moieties of **1**–**4** in C_5_D_5_N.

Position		δ_H_		*J* (Hz)	δ_C_	Position		δ_H_		*J* (Hz)	δ_C_
**1**						**2**					
Glc (I)	1′	4.92	d	7.6	99.8	Glc (I)	1′	4.85	d	8.0	99.8
	2′	4.17	dd	7.9, 7.6	78.9		2′	4.47	dd	9.4, 8.0	79.4
	3′	4.57	dd	7.9, 7.6	79.8		3′	4.33	dd	9.4, 8.1	74.2
	4′	4.58	dd	7.9, 7.9	72.5		4′	4.12	dd	8.1, 8.1	79.4
	5′	4.20	m		78.5		5′	3.72	br d	8.1	77.9
	6′	4.62	br d	12.4	60.9		6′	4.79	br d	11.9	60.5
		4.33	br d	12.4				4.35	br d	11.9	
Rha	1	6.07	br s		102.4	Rha	1	5.94	br s		102.6
	2	4.75	br d	3.4	72.4		2	4.83	br d	3.6	72.3
	3	4.53	dd	9.2, 3.4	72.8		3	4.51	dd	9.7, 3.6	72.7
	4	4.35	dd	9.2, 9.2	73.8		4	4.56	dd	9.7, 9.7	73.7
	5	4.86	dq	9.2, 6.3	70.0		5	4.82	dq	9.7, 6.3	70.0
	6	1.76	d	6.3	18.6		6	1.75	d	6.3	18.6
Ara	1	5.56	d	5.4	103.2	Glc (II)	1′′	5.49	d	7.8	103.9
	2	4.31	dd	6.6, 5.4	73.0		2′′	4.15	dd	8.5, 7.8	74.8
	3	4.19	dd	7.9, 6.6	75.5		3′′	4.24	dd	8.8, 8.5	78.5
	4	4.21	m		70.4		4′′	4.20	dd	8.8, 8.7	71.6
	5	4.67	dd	12.0, 3.5	65.6		5′′	3.84	br d	8.8	78.5
		3.69	dd	12.0, 4.1			6′′	4.40	m	(2H)	61.4
Glc (II)	1′′	5.39	d	7.8	103.0	Ara	1	5.74	d	1.2	102.3
	2′′	4.10	dd	7.9, 7.8	74.9		2	4.72	dd	4.2, 1.2	71.0
	3′′	4.19	dd	7.9, 7.7	77.9		3	4.35	dd	4.2, 4.2	73.3
	4′′	4.25	dd	7.7, 7.1	71.6		4	4.65	m		65.6
	5′′	3.87	m		78.4		5	5.02	dd	11.3. 10.5	62.1
	6′′	4.45	br d	12.8	62.0			3.77	dd	11.3. 4.2	
		4.35	overlapping								
Glc (III)	1′′′	4.82	d	7.8	104.9	Glc (III)	1′′′	4.81	d	7.8	104.9
	2′′′	4.05	dd	8.0, 7.8	75.2		2′′′	4.03	dd	8.3, 7.8	75.1
	3′′′	4.24	dd	8.0, 7.7	78.5		3′′′	4.26	dd	9.1, 8.3	78.6
	4′′′	4.22	dd	8.9, 7.7	71.7		4′′′	4.22	dd	9.1, 9.1	71.7
	5′′′	3.96	m		78.6		5′′′	3.95	br d	9.1	78.7
	6′′′	4.58	br d	12.9	62.8		6′′′	4.52	br d	12.9	62.8
		4.38	br d	12.9				4.39	br d	12.9	
**3**						**4**					
Glc (I)	1′	4.91	d	7.6	99.8	Glc (I)	1′	4.85	d	7.7	99.7
	2′	4.15	dd	7.9, 7.6	78.9		2′	4.46	dd	9.5, 7.7	79.3
	3′	4.55	dd	8.1, 7.9	79.8		3′	4.32	dd	9.5, 7.9	74.3
	4′	4.57	dd	8.1, 8.1	72.6		4′	4.11	dd	7.9, 7.9	79.4
	5′	4.19	m		78.7		5′	3.71	m		77.9
	6′	4.59	br d	12.7	61.4		6′	4.78	br d	11.7	60.4
		4.33	br d	12.7				4.33	br d	11.7	
Rha	1	6.05	br s		102.4	Rha	1	5.92	br s		102.6
	2	4.77	br d	3.5	72.3		2	4.78	br d	3.8	72.4
	3	4.52	dd	9.6, 3.5	72.8		3	4.49	dd	9.7, 3.8	72.7
	4	4.34	dd	9.6, 9.2	73.8		4	4.55	dd	9.8, 9.7	73.7
	5	4.81	dq	9.2, 6.3	70.0		5	4.80	dd	9.8, 6.2	70.0
	6	1.75	d	6.3	18.6		6	1.73	d	6.2	18.6
Ara	1	5.55	d	5.8	103.2	Glc (II)	1′′	5.47	d	7.9	103.9
	2	4.29	dd	6.5, 5.8	73.0		2′′	4.13	dd	8.5, 7.9	74.7
	3	4.21	dd	8.0, 6.5	75.5		3′′	4.24	dd	9.5, 8.5	78.4
	4	4.23	m		70.4		4′′	4.22	dd	9.5, 9.5	71.7
	5	4.66	dd	12.2, 3.9	65.5		5′′	3.82	m		78.5
		3.70	br d	12.2			6′′	4.41	m	(2H)	61.4
Glc (II)	1′′	5.37	d	7.9	103.0	Ara	1	5.72	br s		102.2
	2′′	4.09	dd	7.9, 7.4	74.9		2	4.70	dd	4.6, 1.7	70.9
	3′′	4.19	dd	7.9, 7.4	77.9		3	4.54	overlapping		73.2
	4′′	4.24	dd	7.4, 7.1	72.3		4	4.64	m		65.7
	5′′	3.86	m		78.4		5	4.40	dd	11.5, 11.5	62.1
	6′′	4.34	m	(2H)	62.0			3.76	dd	11.5, 4.5	
Glc (III)	1′′′	4.82	d	7.8	104.9	Glc (III)	1′′′	4.83	d	7.9	104.9
	2′′′	4.05	dd	8.1, 7.8	75.1		2′′′	4.03	dd	7.9, 7.7	75.0
	3′′′	4.24	dd	8.1, 7.8	78.4		3′′′	4.26	dd	9.4, 7.7	78.6
	4′′′	4.22	dd	8.1, 7.8	71.7		4′′′	4.21	dd	9.4, 9.4	71.7
	5′′′	3.96	m		78.6		5′′′	3.97	m		78.7
	6′′′	4.58	br d	12.9	62.8		6′′′	4.56	br d	11.9	62.8
		4.38	br d	12.9				4.40	overlapping		

Compound **2** had the same molecular formula as **1** of C_56_H_92_O_27_, based on the HR-ESI-TOF-MS and ^13^C-NMR (56 carbon signals) data. Two singlet signals for tertiary methyl groups at δ_H_ 1.04 and 0.89, two doublet signals for secondary methyl groups at δ_H_ 1.33 (d, *J* = 6.9 Hz) and 1.00 (d, *J* = 6.8 Hz), and five signals for anomeric protons at δ_H_ 5.94 (br s), 5.74 (d, *J* = 1.2 Hz), 5.49 (d, *J* = 7.8 Hz), 4.85 (d, *J* = 8.0 Hz), and 4.81 (d, *J* = 7.8 Hz) were observed in the ^1^H-NMR spectrum of **2**. The ^13^C-NMR spectrum contained a signal for an acetal carbon at δ_C_ 110.6, and signals for four steroid methyl groups at δ_C_ 19.3, 17.4, 16.4, and 16.4. These spectroscopic data for **2** were analogous to those of **1**, and suggested that **2** shared the same fundamental furostanol skeleton as **1**. Acid hydrolysis of **2** gave **1a**, l-arabinose, d-glucose, and l-rhamnose. Although the ^1^H- and ^13^C-NMR spectra of **2** indicated that the sugar moieties of **2** also consisted of two terminal β-d-glucopyranosyl units (Glc (II) and Glc (III)), a terminal α-l-arabinopyranosyl unit (Ara), a terminal α-l-rhamnopyranosyl unit (Rha), and a 2,3,4-trisubstituted inner β-d-glucopyranosyl moiety (Glc (I)), it was assumed that the linkage positions of the terminal sugar units to the inner Glc (I) moiety and the conformation of the Ara unit were different from those of **1**. The ^13^C-NMR chemical shifts of C-1 to C-5 for Ara were assigned as δ_C_ 102.3, 71.0, 73.3, 65.6, and 62.1, respectively, by combining the ^1^H-^1^H COSY, HMQC, and HSQC-TOCSY spectra. Furthermore, the anomeric proton of Ara at δ_H_ 5.74 (d, *J* = 1.2 Hz) exhibited three-bond coupled strong HMBC correlations with the C-3 and C-5 carbons. These spectral features were consistent with the ^1^C_4_ conformation of Ara with an α-orientation of the anomeric center [11]. In the HMBC spectrum, correlation peaks between the H-1 of Ara at δ_H_ 5.74 and C-4′ of Glc (I) at δ_C_ 79.4, between H-1′′ of Glc (II) at δ_H_ 5.49 and C-3′ of Glc (I) at δ_C_ 74.2, between H-1 of Rha at δ_H_ 5.94 and C-2′ of Glc (I) at δ_C_ 79.4, and between H-1′ of Glc (I) at δ_H_ 4.85 and C-3 of the aglycone at δ_C_ 78.0 showed that Glc (II) and Ara were attached to C-3′ and C-4′ of Glc (I), respectively, in **2**, which was different from **1**. A β-d-glucopyranosyl group (Glc (III)) linkage to the C-26 hydroxy group of the aglycone was ascertained by the HMBC correlation between the H-1′′′ of Glc (III) at δ_H_ 4.81 (d, *J* = 7.8 Hz) and C-26 of the aglycone at δ_C_ 75.2. The NOE correlations between the signals of the H-20 proton at δ_H_ 2.22 and the H_2_-23 protons at δ_H_ 2.04 (2H) confirmed the C-22α configuration. Thus, **2** was elucidated as (25*R*)-26-[(β-d-glucopyranosyl)oxy]-22α-hydroxyfurost-5-*en*-3β-yl *O*-α-l-arabinopyranosyl-(1→4)-*O*-[β-d-glucopyranosyl-(1→3)]-*O*-[α-l-rhamnopyranosyl-(1→2)]-β-d-glucopyranoside.

Compound **3** was analyzed as C_56_H_90_O_26_ based on the HR-ESI-TOF-MS (*m*/*z* 1201.5615 [M + Na]^+^) and ^13^C-NMR (56 carbon signals) data. This molecular formula was smaller than that of **1** by 18.0059 (H_2_O). The ^1^H- and ^13^C-NMR spectral features of **3** were similar to those of **1**; however, the Me-21 doublet signal at δ_H_ 1.34 (d, *J* = 6.9 Hz) in the ^1^H-NMR spectrum of **1** was replaced by a methyl singlet signal at δ_H_ 1.64 in that of **3**, and an olefinic functionality (δ_C_ 152.3 and 103.6) was observed in addition to the 5(6)-ene group in the ^13^C-NMR spectrum of **3**. These spectroscopic data and the HMBC correlations from H-17 at δ_H_ 2.45, H_2_-23 at δ_H_ 2.22, and Me-21 at δ_H_ 1.64, to C-22 at δ_C_ 152.3 and C-20 at δ_C_ 103.6 indicated that **3** was the corresponding Δ^20(22)^-pseudo-furostanol glycoside of **1**. This was supported by all other spectroscopic data and the results of acid hydrolysis. The structure of **3** was assigned as (25*R*)-26-[(β-d-glucopyranosyl)oxy]-furosta-5,20(22)-dien-3β-yl *O*-α-l-arabinopyranosyl-(1→3)-*O*-[β-d-glucopyranosyl-(1→4)]-*O*-[α-l-rhamnopyranosyl-(1→2)]-β-d-glucopyranoside.

Compound **4** was shown to have a molecular formula of C_56_H_90_O_26_ by the HR-ESI-TOF-MS (*m*/*z* 1201.5653 [M + Na]^+^, calcd. 1201.5618) and ^13^C-NMR (56 carbon signals) data, and its aglycone was identified as the same Δ^20(22)^-pseudo-furostanol derivative as **3**, based on its spectroscopic data. Further spectroscopic analysis and acid hydrolysis of **4** indicated that the sugar moieties attached to C-3 and C-26 of the aglycone were in agreement with those of **2**, in which the terminal α-l-arabinopyranosyl group was present as an unusual ^1^C_4_ conformation. It is notable that the ^1^C_4_ α-l-arabinopyranosyl moiety in **4** was converted to the ^4^C_1_ moiety on peracetylation (**4a**). Therefore, **4** was the corresponding Δ^20(22)^-pseudo-furostanol glycoside of **2**, (25*R*)-26-[(β-d-glucopyranosyl)oxy]-furosta-5,20 (22)-dien-3β-yl *O*-α-l-arabinopyranosyl-(1→4)-*O*-[β-d-glucopyranosyl-(1→3)]-*O*-[α-l-rhamnopyranosyl-(1→2)]-β-d-glucopyranoside.

The isolated compounds **1**–**4** were evaluated for their cytotoxic activity against HL-60 cells. They were not cytotoxic to HL-60 cells at sample concentrations up to 15 μM.

## 3. Experimental Section

### 3.1. General Experimental Procedures

Optical rotations were measured by using an automatic digital polarimeter (P-1030, Jasco, Tokyo, Japan). IR spectra were recorded on a spectrophotometer (FT-IR 620, Jasco, Tokyo, Japan). ^1^H-NMR spectra were obtained at 500 MHz by using standard Bruker pulse programs at 300 K (DRX-500, Bruker, Karlsruhe, Germany). Chemical shifts are given as δ values relative to tetramethylsilane (TMS, Wako, Osaka, Japan) used as an internal standard. HR-ESI-TOF-MS data were recorded on an LCT mass spectrometer (Waters-Micromass, Manchester, UK). Diaion HP-20 (50 mesh, Mitsubishi-Chemical, Tokyo, Japan), BW-300 silica gel (200-300 mesh, Fuji Silysia Chemical, Aichi, Japan), and COSMOSIL 75C_18_-OPN ODS silica gel (75 μM, Nacalai Tesque, Kyoto, Japan) were used for CC. TLC was carried out on precoated silica gel 60 F_254_ (0.25 mm thick, Merck, Darmstadt, Germany) and RP_18_ F_254_S plates (0.25 mm thick, Merck), and the compounds were visualized by spraying the plates with 10% H_2_SO_4_ (aq.) and then heating. HPLC was performed with a system consisting of a CCPM pump (Tosoh, Tokyo, Japan), an RI-8021 detector (Tosoh), and a Rheodyne injection port (Rohnert Park, CA, USA). A TSK gel ODS-100Z column (10 mm i.d. × 250 mm, 5 μm, Tosoh) was used for preparative HPLC. NMR spectra are available in the Supplementary materials.

### 3.2. Plant Material

The *L. pumilum* bulbs were obtained from the Sakata Seed Corporation (Yokohama, Japan) in 2008 and were identified by Dr. Yutaka Sashida, professor emeritus of Tokyo University of Pharmacy and Life Sciences. We have retained a voucher specimen in our laboratory (KS-2008-002).

### 3.3. Extraction and Isolation

The bulbs of *L. pumilum* (1.4 kg fr. weight) were extracted with MeOH under reflux for 6 h. After removing the solvent, the MeOH extract (65 g) was passed through a Diaion HP-20 column (2000 g, 8.5 cm i.d. × 60 cm) and successively eluted with MeOH–H_2_O (3:7, 1:1), MeOH, EtOH, and EtOAc (6 L of each eluent). CC of the MeOH-eluted fraction (1.8 g) on silica gel (2000 g, 8.0 cm i.d. × 30 cm), eluted with a stepwise gradient mixture of CHCl_3_–MeOH–H_2_O (7:4:1) and finally with MeOH, provided 6 fractions (M-1 to M-6). Fraction M-4 (0.5 g) was chromatographed on ODS silica gel (700 g, 4.0 cm i.d. × 25 cm) eluted with MeOH–H_2_O (2:1) and finally with MeOH, provided 9 fractions (M-4-1–M-4-9). Fraction M-4-5 (148 mg) was separated by HPLC (1.0 cm i.d. × 25 cm) using MeCN–H_2_O (1:3) to afford **3** (14.3 mg) and **4** (15.0 mg). Fraction M-4-7 (94 mg) was separated by HPLC (1.0 cm i.d. × 25 cm) using MeCN–H_2_O (1:2) to afford **1** (20.6 mg) and **2** (20.6 mg).

### 3.4. Acid Hydrolysis of ***1***, ***2***, ***3***, or ***4***

A solution of **1** (14.5 mg), **2** (14.5 mg), **3** (5.0 mg), or **4** (5.0 mg) in 1 M HCl in dioxane–H_2_O (1:1; 2.0 mL) were heated at 90 °C for 2 h under an Ar atmosphere, respectively. The reaction mixture was neturalized by passing it through an Amberlite column (IRA-96SB, Organo, Tokyo, Japan; 16–50 mesh, 50 g, 1.5 cm i.d. × 15 cm). The mixture was then eluted through a Diaion HP-20 column (50 g, 1.5 cm i.d. × 15 cm) with MeOH–H_2_O (3:7) and EtOH–Me_2_CO (1:1). The EtOH–Me_2_CO (1:1) fraction was purified by CC with CHCl_3_–MeOH–H_2_O (7:4:1) to give diosgenin (**1a**; 1.9 mg; 0.2 mg; 1.1 mg; and 5.0 mg), respectively. The MeOH–H_2_O (3:7) fraction was analyzed by HPLC under the following conditions: Capcell Pak NH2 UG80 column (4.6 mm i.d. × 25 cm, 5 μm, Shiseido, Tokyo, Japan); mobile phase of MeCN–H_2_O (85:15); detection by refractive index and optical rotation; and a flow rate of 1.0 mL/min. l-Arabinose, d-glucose, and l-rhamnose were identified, respectively, by comparing their retention times (*t*_R_) and optical rotation with those of authentic samples (l-arabinose 7.00 min, positive optical rotation; d-glucose 12.28 min, positive optical rotation; l-rhamnose 6.65 min, negative optical rotation): l-arabinose (6.85 min; 6.89 min; 6.77 min; 8.00 min; positive optical rotation), d-glucose (12.25 min; 12.74 min; 12.33 min; 12.74 min; positive optical rotation), and l-rhamnose (6.56 min; 6.60 min; 6.60 min; 6.97 min; negative optical rotation).

### 3.5. Acetylation of ***4***

A mixture of **4** (8.0 mg) and Ac_2_O (1.0 mL) in pyridine (7.0 mL) was stirred at room temperature for 24 h. The reaction mixture was purified by CC with CHCl_3_–EtOAc (1:1) to give the corresponding peracetylation (**4a**, 3.2 mg).

### 3.6. Data for ***1***–***4*** and ***4a***

*(25R)-26-[(β-d-Glucopyranosyl)oxy]-22α-hydroxyfurost-5-en-3β-yl O-α-L-arabinopyranosyl-(1→3)-O-[β-d-glucopyranosyl-(1→4)]-O-[α-l-rhamnopyranosyl-(1→2)]-β-d-glucopyranoside* (**1**); An amorphous solid; ${\left[\text{α}\right]}_{D}^{25}$ −54.2 (*c* 0.10, MeOH); HR-ESI-TOF-MS (*m*/*z*: 1219.5674 [M + Na]^+^, calcd for C_56_H_92_NaO_27_: 1219.5724); IR ν_max_ (film) cm^−1^: 3388 (OH), 2931 (CH); ^1^H-NMR (500 MHz, C_5_D_5_N): for the aglycone moiety, 5.33 (1H, br s, H-6), 4.96 (1H, br d, *J* = 6.8 Hz, H-16), 3.95 (1H, dd, *J* = 9.5, 7.1 Hz , H-26a), 3.86 (1H, m, *W*_1/2_ = 21.6 Hz, H-3), 3.60 (1H, dd, *J* = 9.5, 7.8 Hz, H-26b), 1.34 (3H, d, *J* = 6.9 Hz, Me-21), 1.04 (3H, s, Me-19), 1.00 (3H, d, *J* = 6.7 Hz, Me-27), 0.89 (3H, s, Me-18). For the sugar moieties, Table 2; ^13^C-NMR (125 MHz, C_5_D_5_N): Table 1 and Table 2.

*(25R)-26-[(β-d-Glucopyranosyl)oxy]-22α-hydroxyfurost-5-en-3β-yl O-α-l-arabinopyranosyl-(1→4)-O-[β-d-glucopyranosyl-(1→3)]-O-[α-l-rhamnopyranosyl-(1→2)]-β-d-glucopyranoside* (**2**); An amorphous solid; ${\left[\text{α}\right]}_{D}^{25}$ −59.6 (*c* 0.10, MeOH); HR-ESI-TOF-MS (*m*/*z*: 1219.5693 [M + Na]^+^, calcd for C_56_H_92_NaO_27_: 1219.5724); IR ν_max_ (film) cm^−1^: 3388 (OH), 2926 (CH). ^1^H-NMR (500 MHz, C_5_D_5_N): for the aglycone moiety, 5.33 (1H, br s, H-6), 4.95 (1H, br d, *J* = 7.5 Hz, H-16), 3.96 (1H, dd, *J* = 8.5, 7.0 Hz , H-26a), 3.84 (1H, overlapping, H-3), 3.59 (1H, dd, *J* = 8.5, 4.0 Hz, H-26b), 1.33 (3H, d, *J* = 6.9 Hz, Me-21), 1.04 (3H, s, Me-19), 1.00 (3H, d, *J* = 6.8 Hz, Me-27), 0.89 (3H, s, Me-18). For the sugar moieties, Table 2; ^13^C-NMR (125 MHz, C_5_D_5_N): Table 1 and Table 2.

*(25R)-26-[(β-d-Glucopyranosyl)oxy]-furosta-5,20(22)-dien-3β-yl O-α-l-arabinopyranosyl-(1→3)-O-[β-d-glucopyranosyl-(1→4)]-O-[α-l-rhamnopyranosyl-(1→2)]-β-d-glucopyranoside* (**3**); An amorphous solid; ${\left[\text{α}\right]}_{D}^{25}$ −42.6 (*c* 0.04, MeOH); HR-ESI-TOF-MS (*m*/*z*: 1201.5615 [M + Na]^+^, calcd for C_56_H_90_NaO_2__6_: 1201.5618); IR ν_max_ (film) cm^−1^: 3326 (OH), 2925 (CH); ^1^H-NMR (500 MHz, C_5_D_5_N): for the aglycone moiety, 5.33 (1H, br d, *J* = 4.4 Hz, H-6), 4.75 (1H, m, H-16), 3.95 (1H, dd, *J* = 9.0, 6.9 Hz, H-26b), 3.84 (1H, m, *W*_1/2_ = 25.3 Hz, H-3), 3.66 (1H, br d, *J* = 9.0 Hz, H-26a), 1.64 (3H, s, Me-21), 1.05 (3H, s, Me-19), 1.02 (3H, d, *J* = 6.7 Hz, Me-27), 0.73 (3H, s, Me-18). For the sugar moieties, Table 2; ^13^C-NMR (125 MHz, C_5_D_5_N): Table 1 and Table 2.

*(25R)-26-[(β-d-Glucopyranosyl)oxy]-furosta-5,20(22)-dien-3β-yl O-α-l-arabinopyranosyl-(1→4)-O-[β-d-glucopyranosyl-(1→3)]-O-[α-l-rhamnopyranosyl-(1→2)]-β-d-glucopyranoside* (**4**); An amorphous solid; ${\left[\text{α}\right]}_{D}^{25}$ −51.7 (*c* 0.03, MeOH); HR-ESI-TOF-MS (*m*/*z*: 1201.5653 [M + Na]^+^ calcd for C_56_H_90_NaO_26_, 1201.5618); IR ν_max_ (film) cm^−1^: 3357 (OH), 2925 (CH); ^1^H-NMR (500 MHz, C_5_D_5_N): for the aglycone moiety, 5.33 (1H, br d, *J* = 4.3 Hz, H-6), 4.75 (1H, m, H-16), 3.94 (1H, dd, *J* = 9.0, 7.0 Hz, H-26a), 3.83 (1H, m, *W*_1/2_ = 27.5 Hz, H-3), 3.47 (1H, dd, *J* = 9.0, 6.1 Hz, H-26b), 1.63 (3H, s, Me-21), 1.04 (3H, s, Me-19), 1.01 (3H, d, *J* = 6.7 Hz, Me-27), 0.72 (3H, s, Me-18). For the sugar moieties, Table 2; ^13^C-NMR (125 MHz, C_5_D_5_N): Table 1 and Table 2.

*Compound* **4a**; An amorphous solid; ${\left[\text{α}\right]}_{D}^{25}$ −33.0 (*c* 0.16, MeOH); HR-ESI-TOF-MS (*m*/*z*: 1831.7181 [M + ONa]^+^, calcd for C_86_H_120_NaO_41_: 1831.7203); IR ν_max_ (film) cm^−^^1^: 2960 and 2924 (CH), 1747 (C=O); ^1^H-NMR (500 MHz, C_5_D_5_N): δ_H_ 5.50 (1H, br d, *J* = 1.5 Hz, H-6), 3.90 (1H, m, overlapping, H-3), 3.89 (1H, dd, *J* = 9.4, 5.9 Hz, H-26a), 3.47 (1H, dd, *J* = 9.4, 6.1 Hz, H-26b), 0.94 (3H, d, *J* = 6.6 Hz, Me-27), 1.11 (3H, s, Me-19), 0.78 (3H, s, Me-18), 5.71 (1H, br s, H-1 of Rha), 5.11 (1H, d, *J* = 6.5 Hz, H-1 of Ara), 5.18 (1H, d, *J* = 7.8 Hz, H-1′′ of Glc (II)), 4.92 (1H, d, *J* = 8.0 Hz, H-1′′′ of Glc (III)), 4.89 (1H, d, *J* = 7.7 Hz, H-1′ of Glc (I)), 1.47 (3H, d, *J* = 6.2 Hz, H-6 of Rha), 2.26, 2.24, 2.22, 2.20, 2.16, 2.06, 2.04 × 2, 2.03, 2.02 × 2, 2.00, 1.98 × 2, 1.91 (each 3H, s, Ac × 15); ^13^C-NMR (125 MHz, C_5_D_5_N) : δ_C_ 152.3 (C-22), 140.4 (C-5), 122.3 (C-6), 84.5 (C-16), 78.5 (C-3), 19.3 (C-19), 16.7 (C-27), 14.1 (C-18), 103.7 (C-20), 11.7 (C-21), 101.4 (C-1′′′ of Glc (III)), 101.2 (C-1 of Ara), 99.9 (C-1′′ of Glc (II)), 99.0 (C-1′ of Glc (I)), 97.8 (C-1 of Rha), 78.6 (C-3′ of Glc (I)), 77.6 (C-2′ of Glc (I)), 76.8 (C-4′ of Glc (I)), 63.1 (C-5 of Ara), 63.1 (C-6′ of Glc (I)), 62.4 (C-6′′′ of Glc (III)), 61.6 (C-6′′of Glc (II)), 17.5 (C-6 of Rha).

### 3.7. Cell Culture Assay

Cell growth was measured with an MTT reduction assay, as described in a previous paper [12].

## 4. Conclusions

In conclusion, four new steroidal glycosides (**1**–**4**) were isolated from the bulbs of *L. pumilum*, and they were classified into furostanol glycosides (**1** and **2**) and Δ^20(22)^-pseudo-furostanol glycosides (**3** and **4**). Compounds **1**–**4** are novel steroidal glycosides with a 2,3,4-trisubstituted β-d-glucopyranosyl moiety at C-3 of the aglycone. In general, α-l-arabinopyranosyl groups are stable in a ^4^C_1_ conformation in glycosides, except for those glycosylated at C-2 [13]. In **1** and **3**, the α-l-arabinopyranosyl moiety is linked to C-3 of the inner trisubstituted β-d-glucopyranosyl group and is present as an usual ^4^C_1_ conformation. In contrast, in **2** and **4**, the α-l-arabinopyranosyl moiety, which is attached to C-4 of the inner trisubstituted β-d-glucopyranosyl group, is present as a ^1^C_4_ conformation. It is notable that the ^1^C_4_ α-l-arabinopyranosyl moiety in **4** was converted to the ^4^C_1_ moiety on peracetylation (**4a**). These interesting steric behaviors of arabinopyranose cannot be explained by steric hindrance only. In a recent study, there are reports of the inner substituted ^1^C_4_ conformation of α-l-arabinopyranosyl moiety in triterpene glycoside [14,15]. Many more examples and data need to be collected in future work.

## Supplementary Materials

Supplementary materials can be accessed at: http://www.mdpi.com/1420-3049/20/09/16255/s1.
