# Supplementary material for: Novel Steroidal Glycosides from the Bulbs of Lilium pumilum
Source: Molecules. 2015 Sep 8;20(9):16255–65. doi: 10.3390/molecules200916255 (PMC6331790; doi:10.3390/molecules200916255)
Supplement: Supplementary file 1 [file molecules-20-16255-s001.pdf]

Supplementary Materials

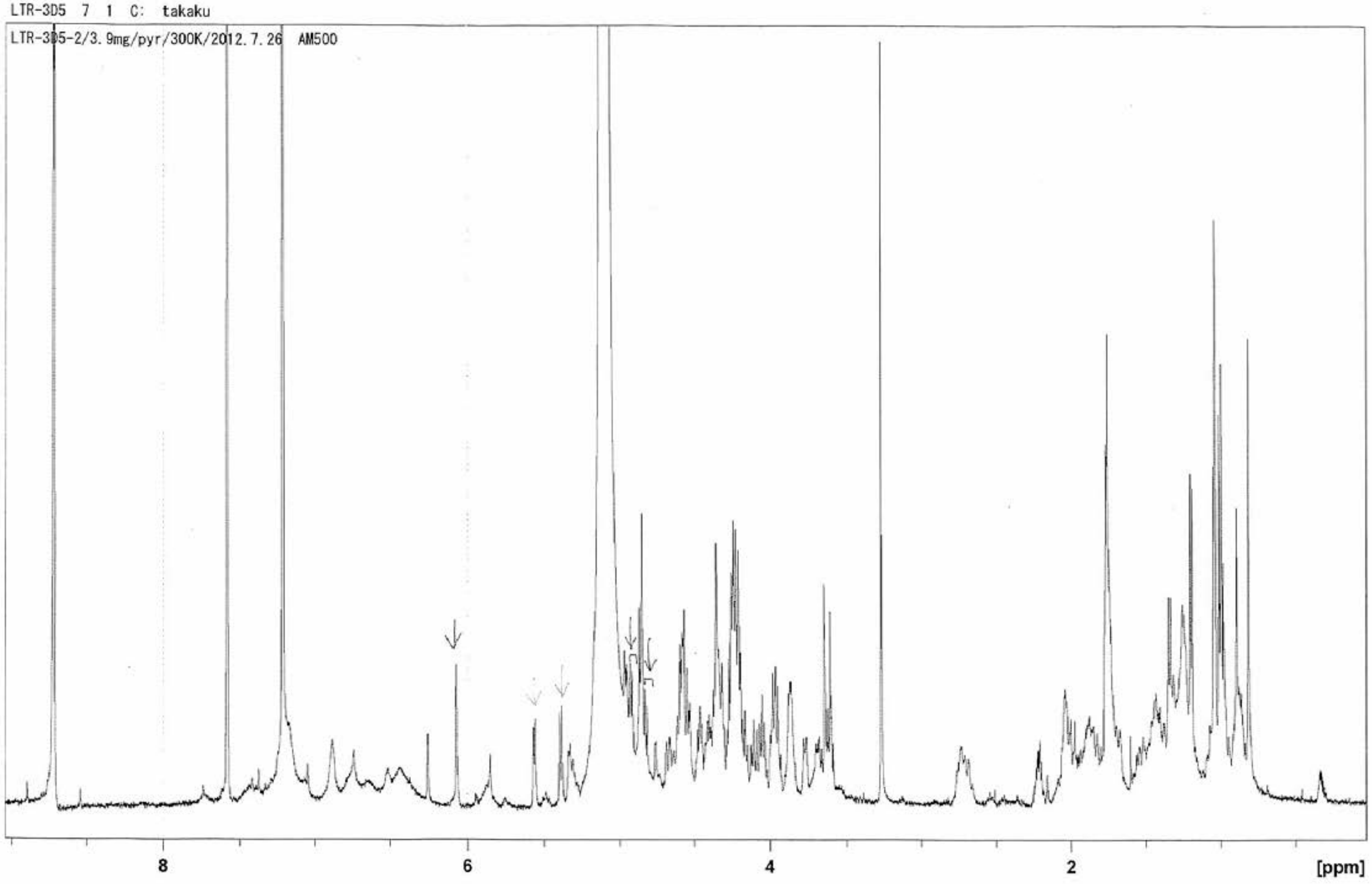

Figure S1. ^{1}H-NMR spectrum of **1**.

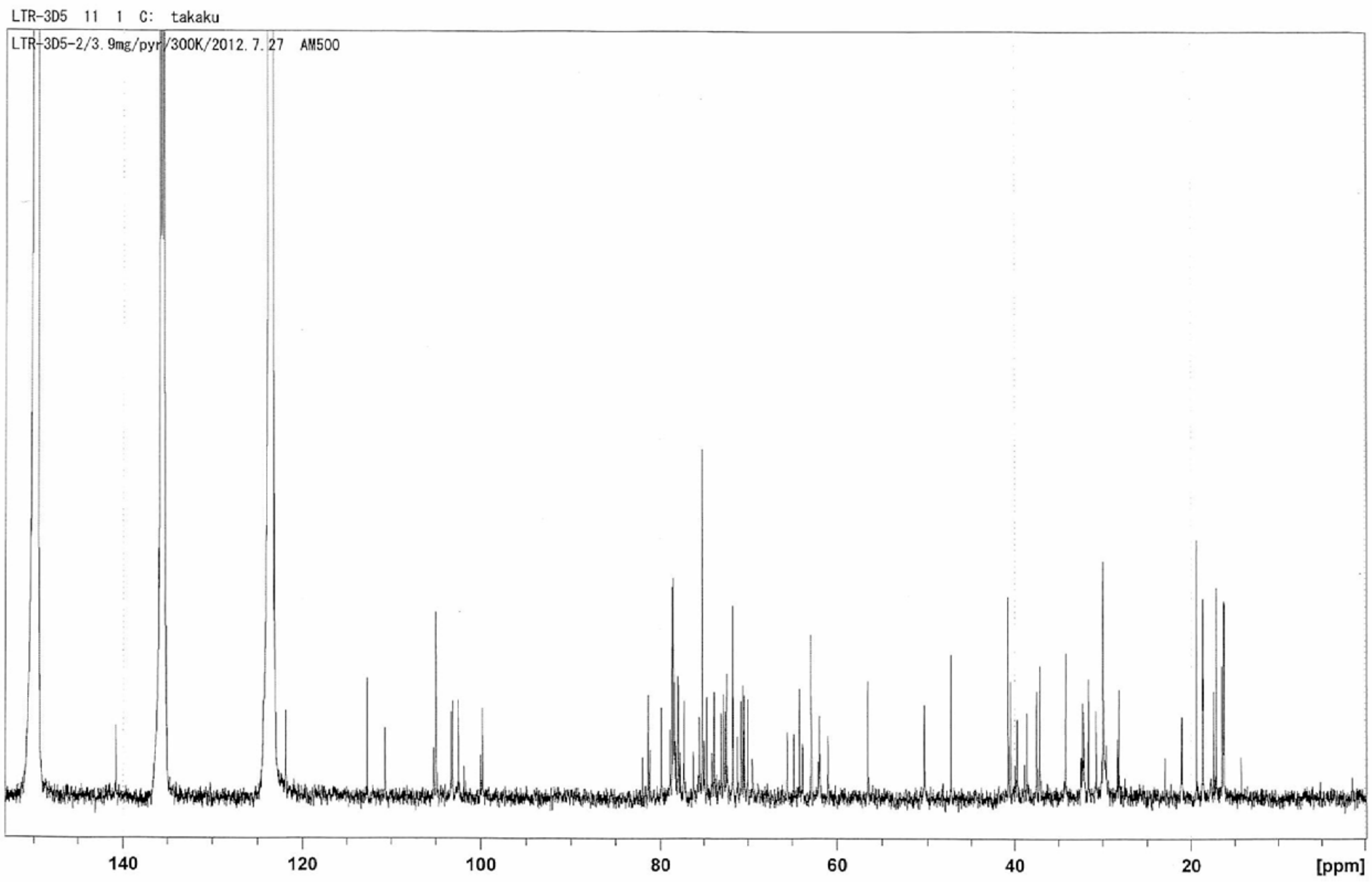

Figure S2. ^{13}C-NMR spectrum of **1**.

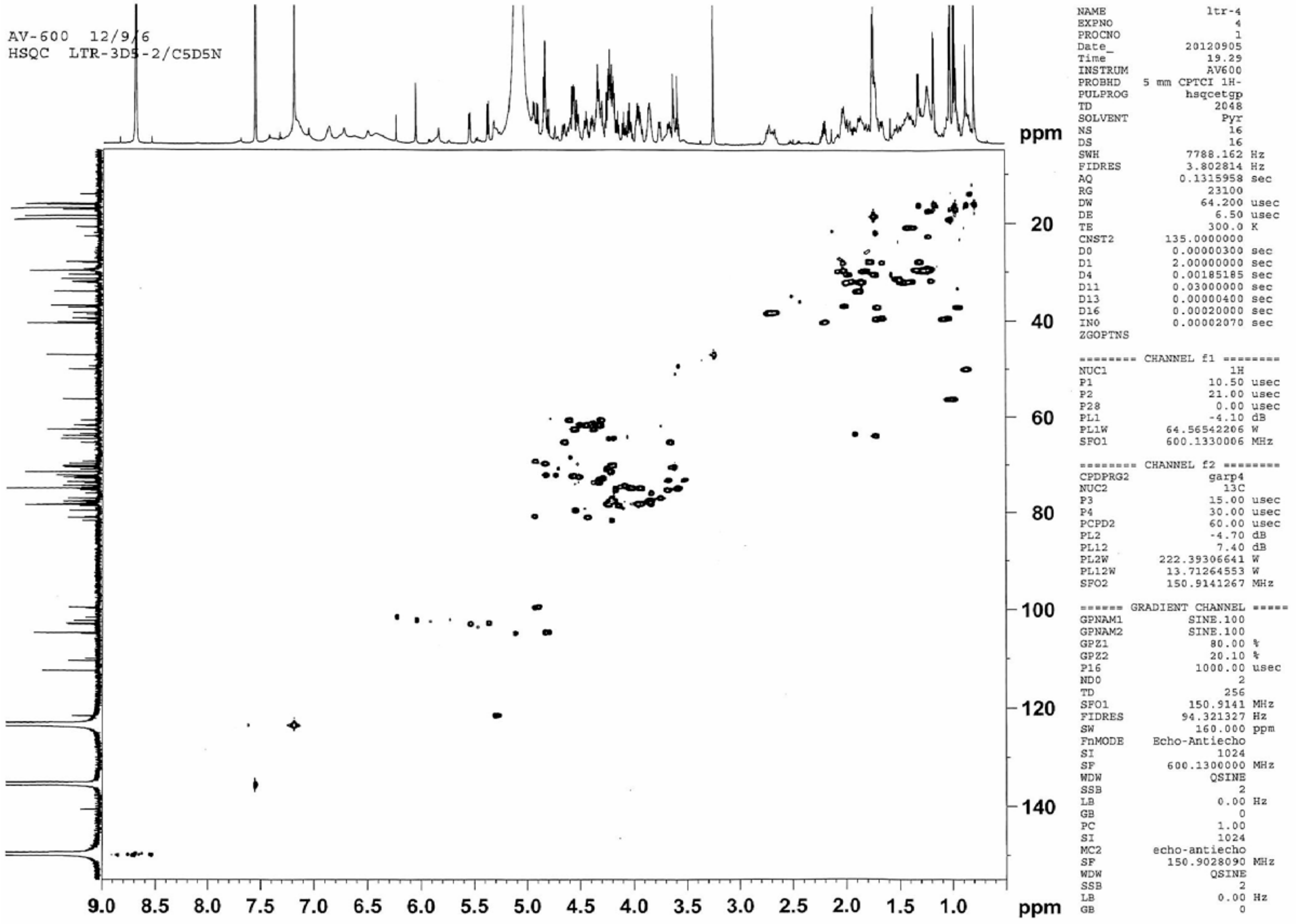

Figure S3. HMQC of **1**.

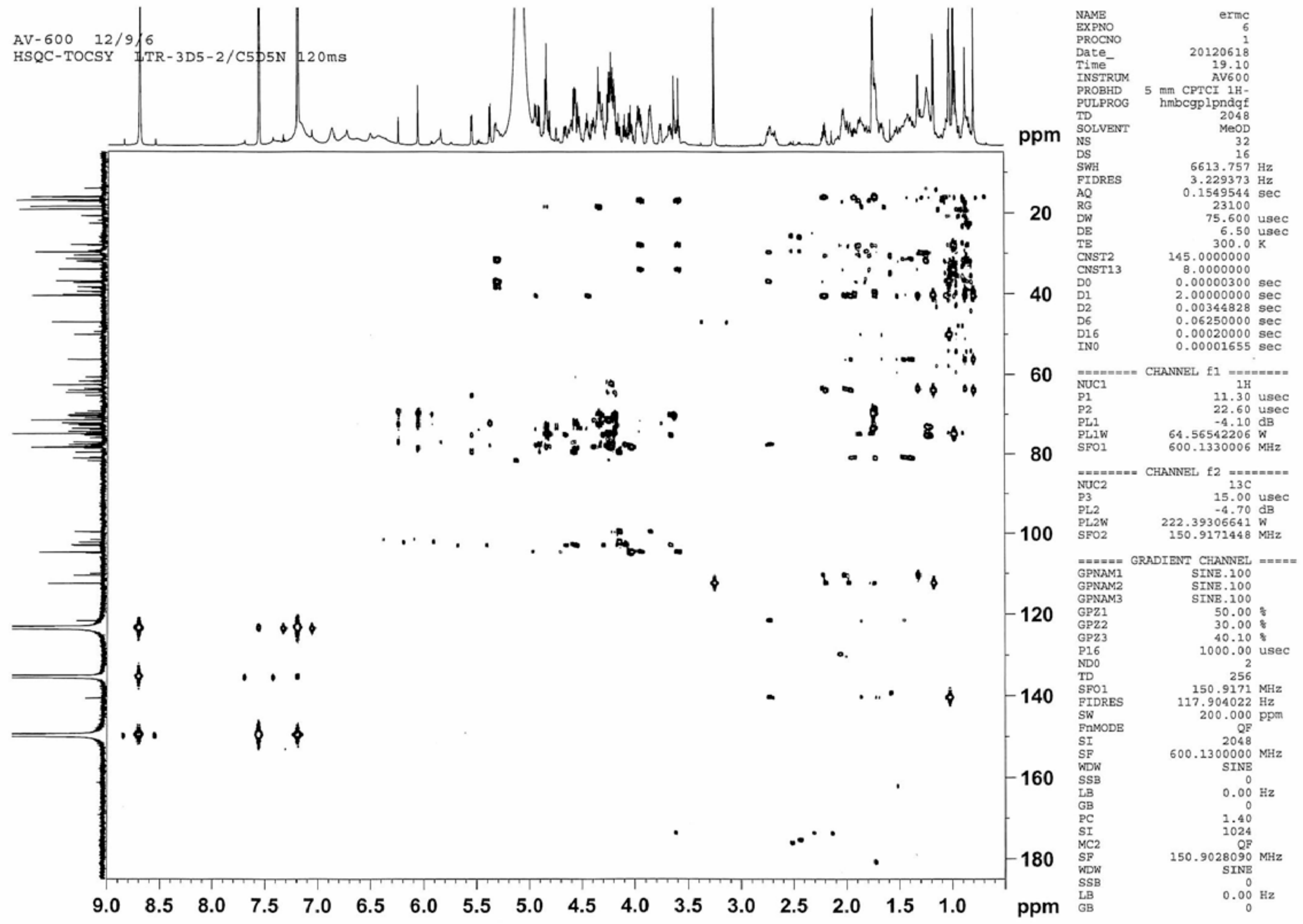

Figure S4. HMBC of **1**.

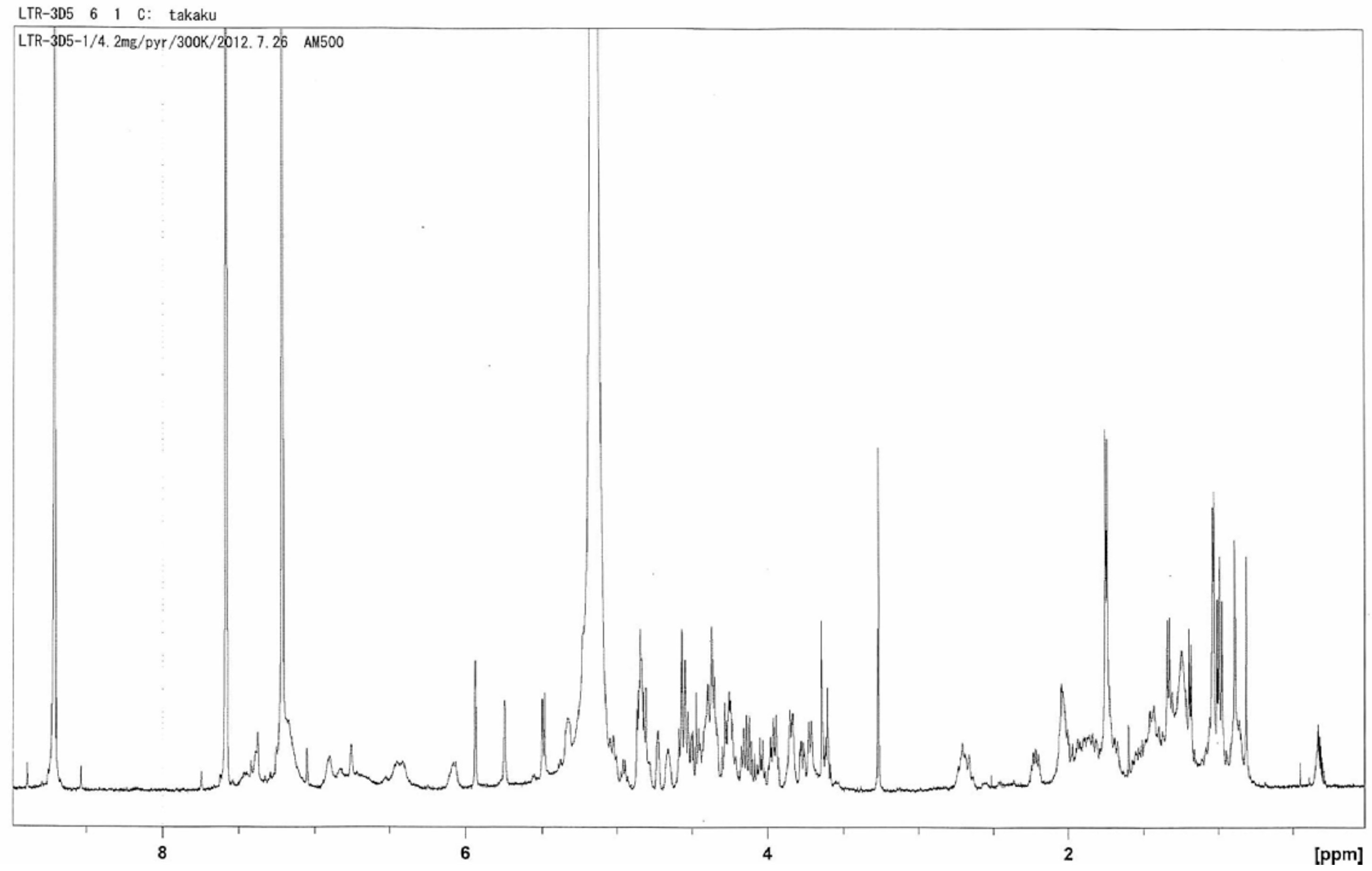

Figure S5. ¹H-NMR spectrum of **2**.

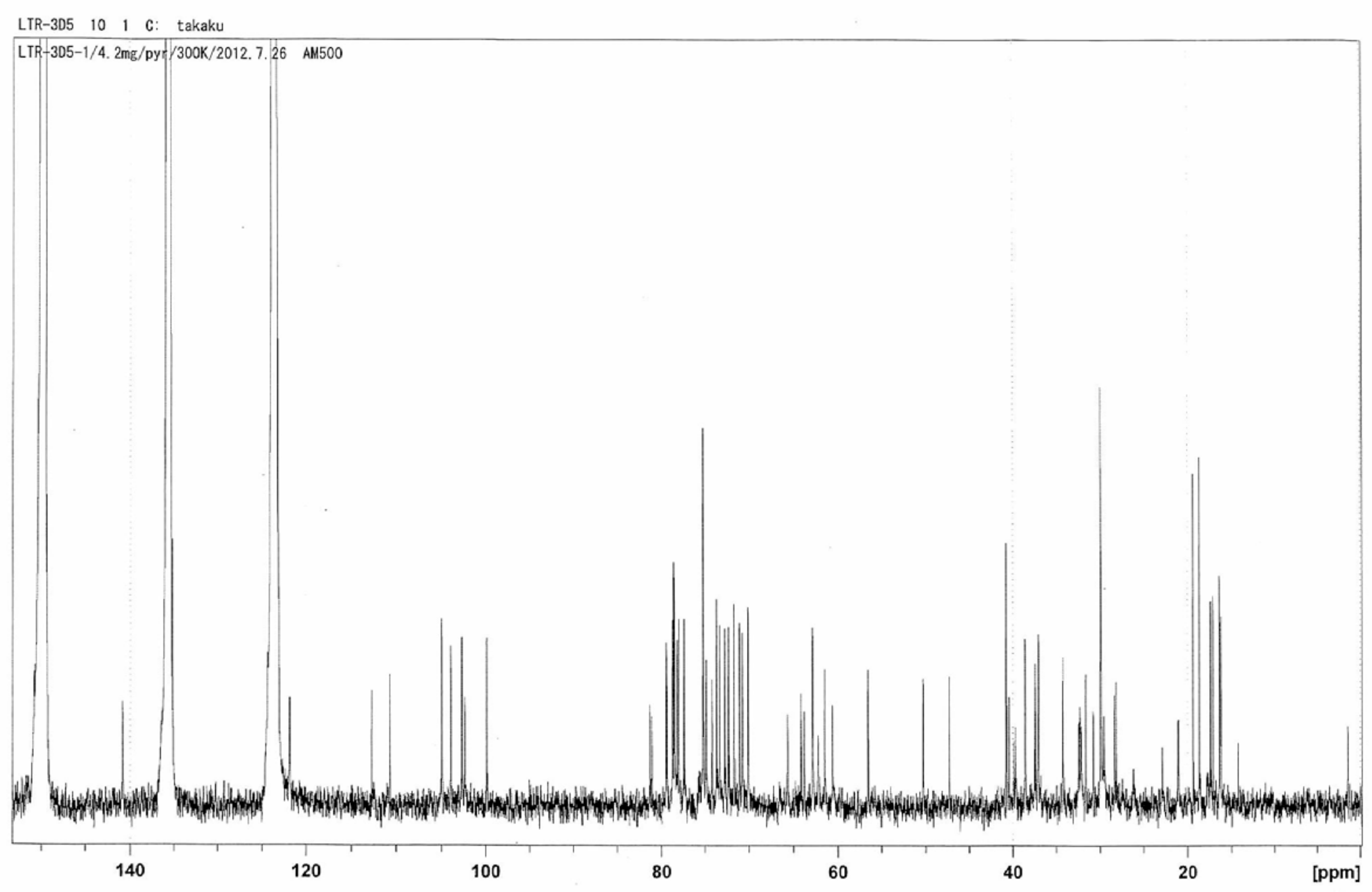

Figure S6. ^{13}C-NMR spectrum of **2**.

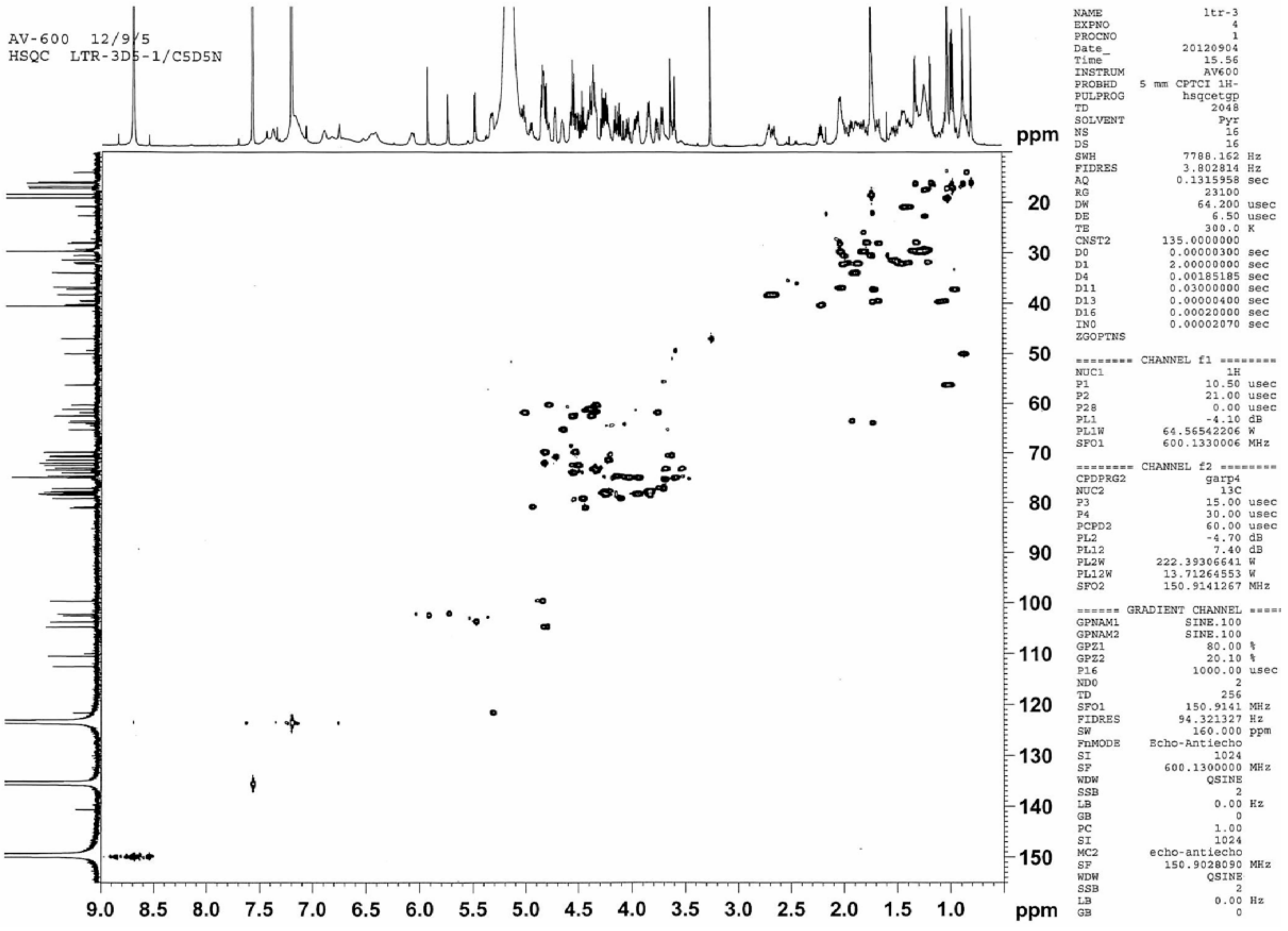

Figure S7. HMQC of **2**.

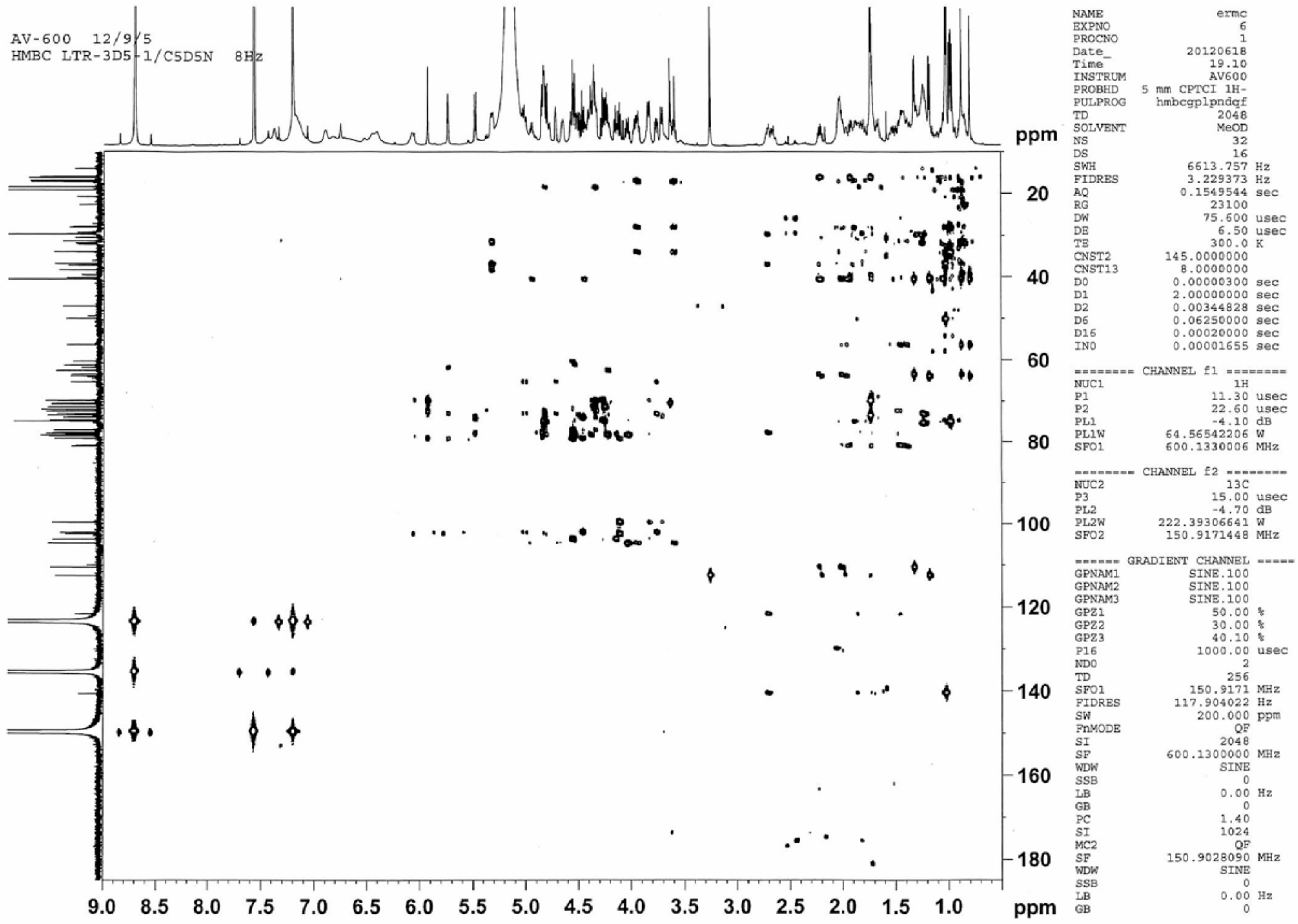

Figure S8. HMBC of **2**.

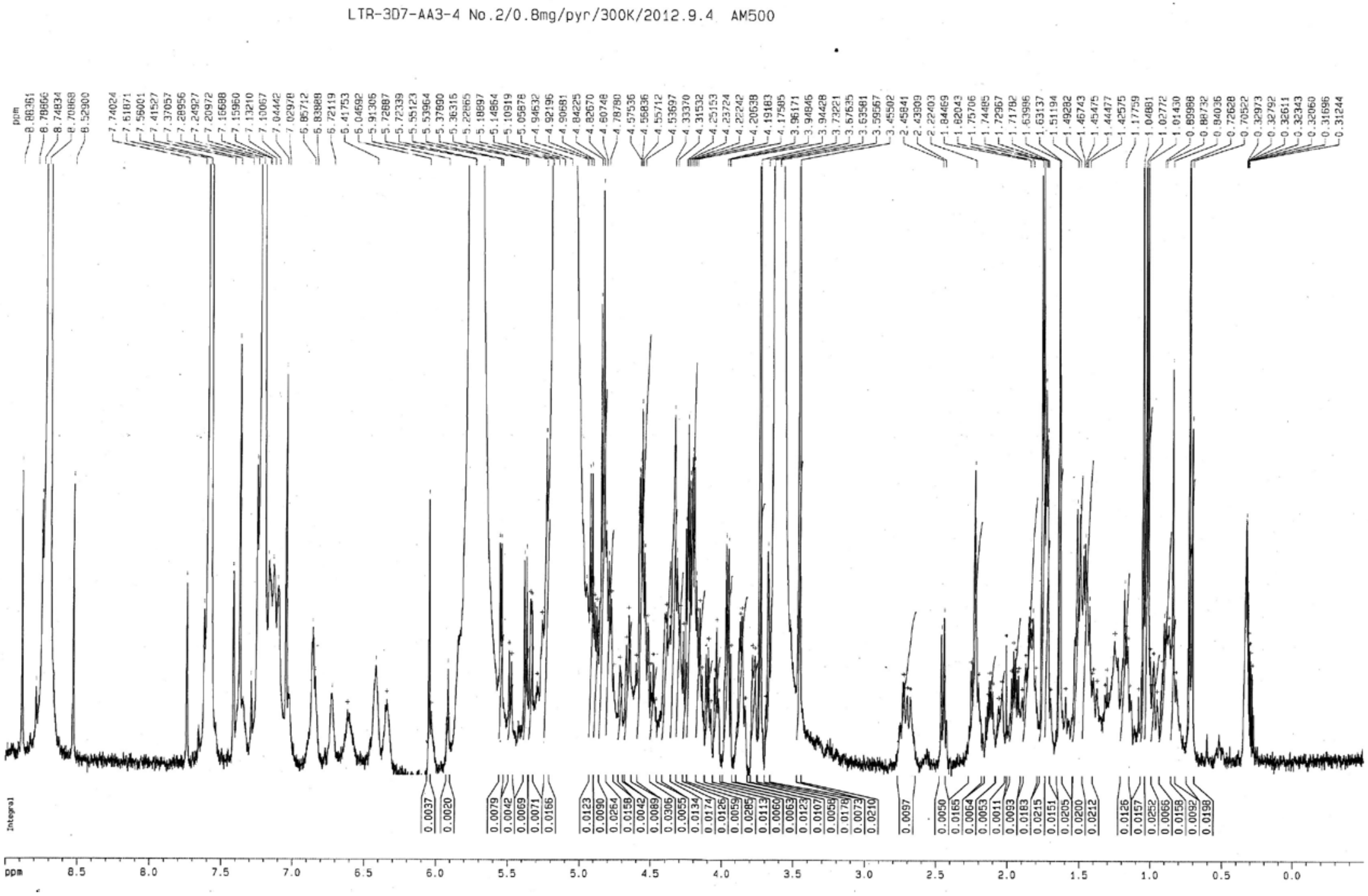

Figure S9. ^{1}H-NMR spectrum of **3**.

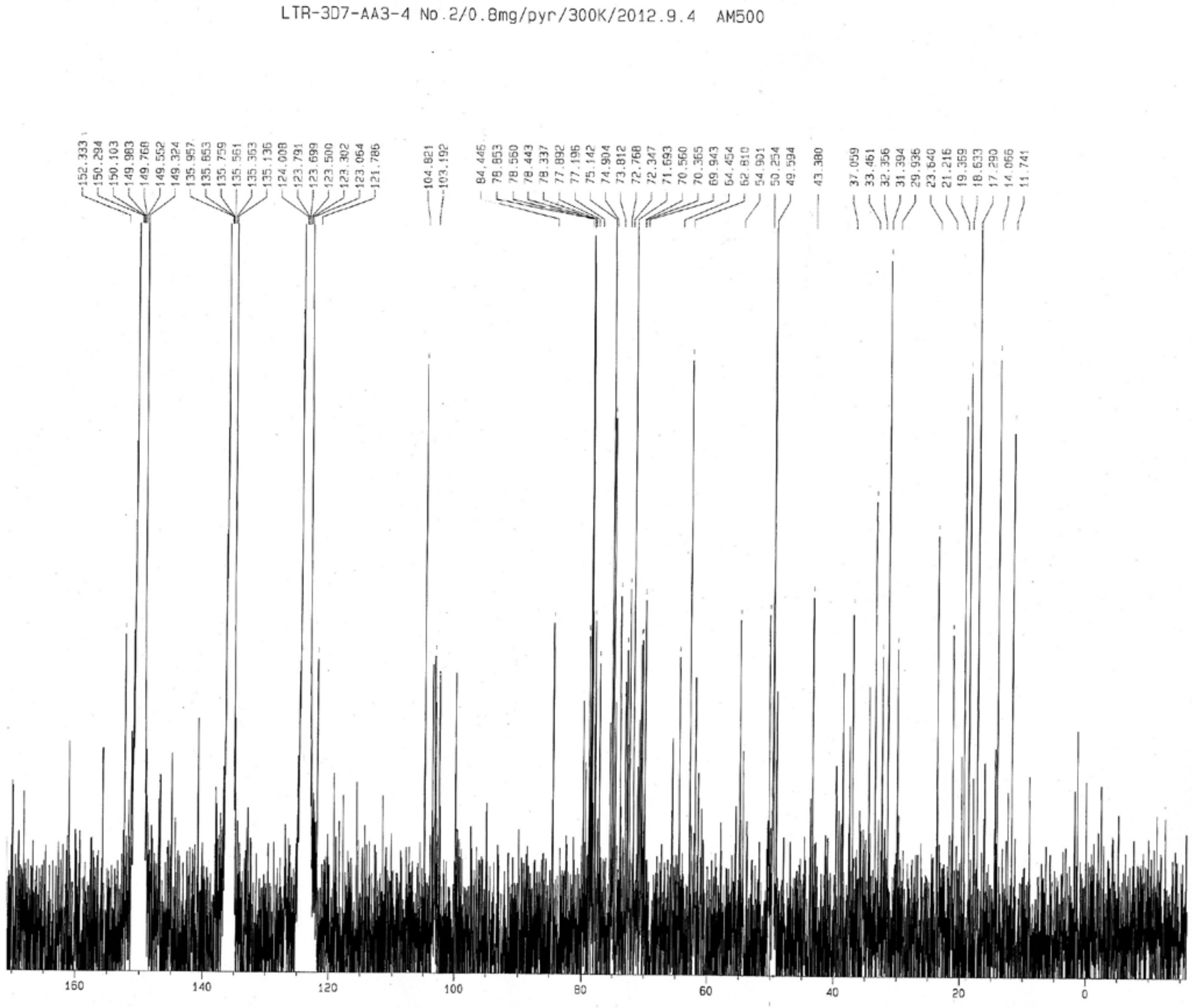

Figure S10. ^{13}C-NMR spectrum of **3**.

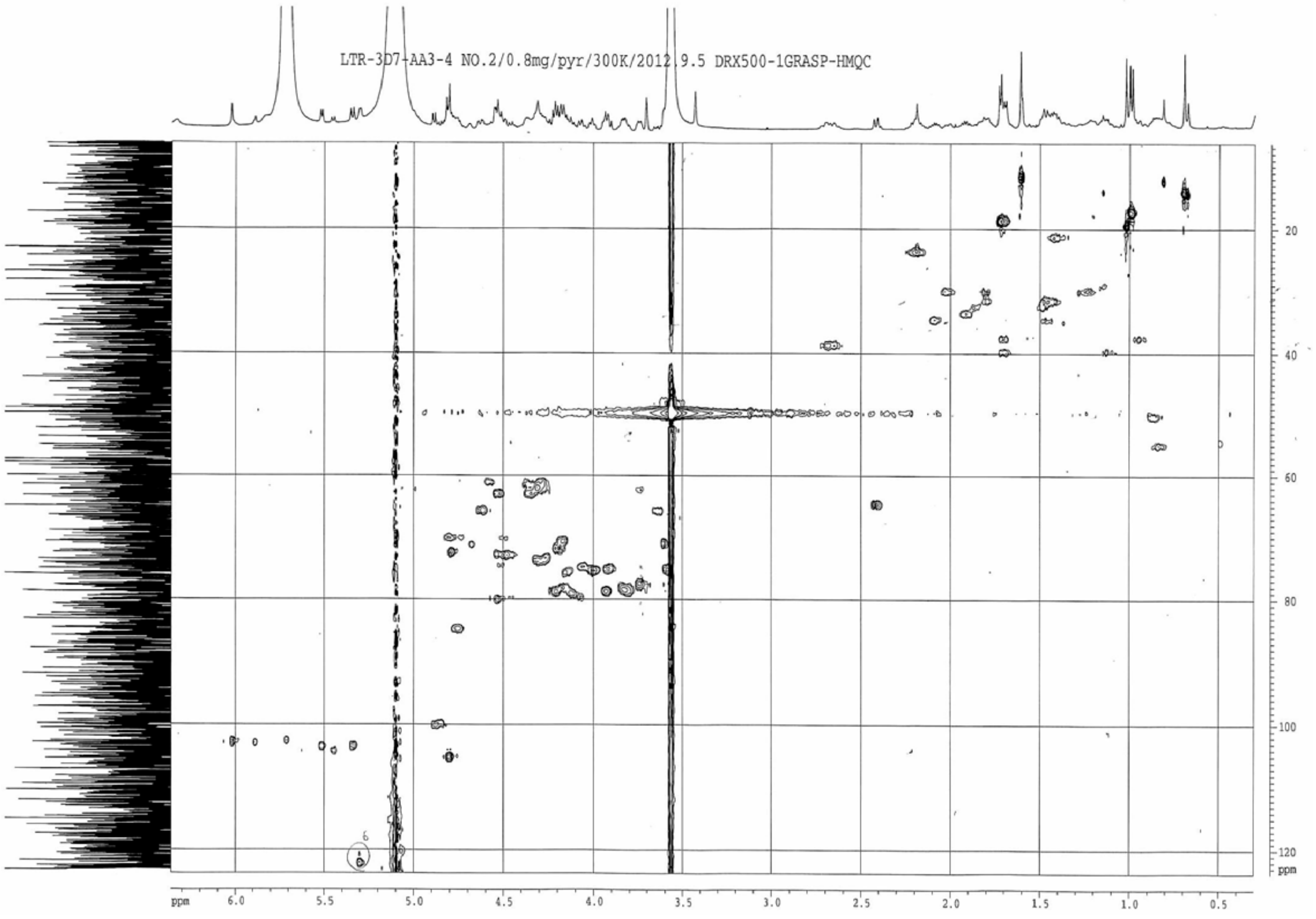

Figure S11. HMQC of **3**.

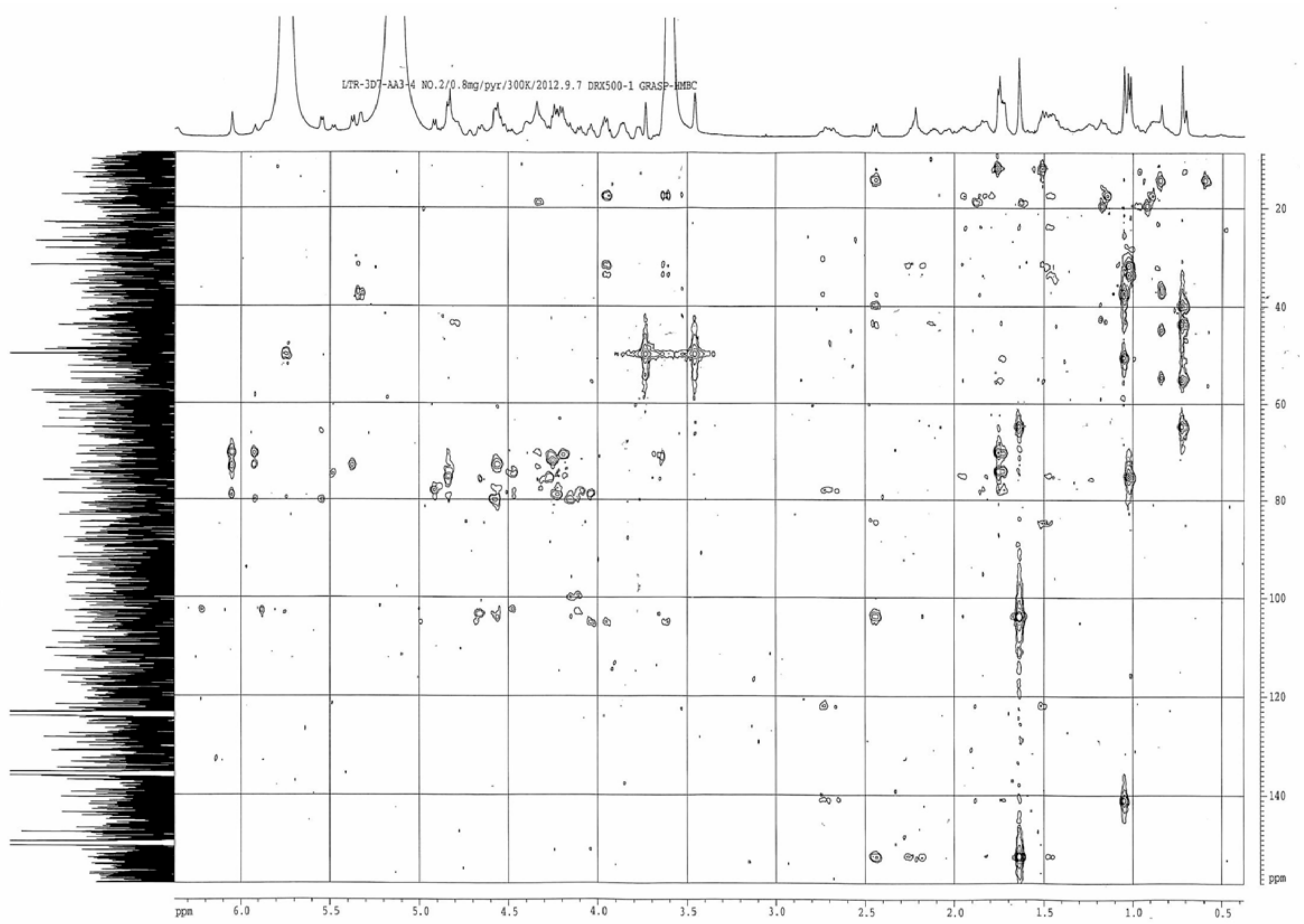

Figure S12. HMBC of **3**.

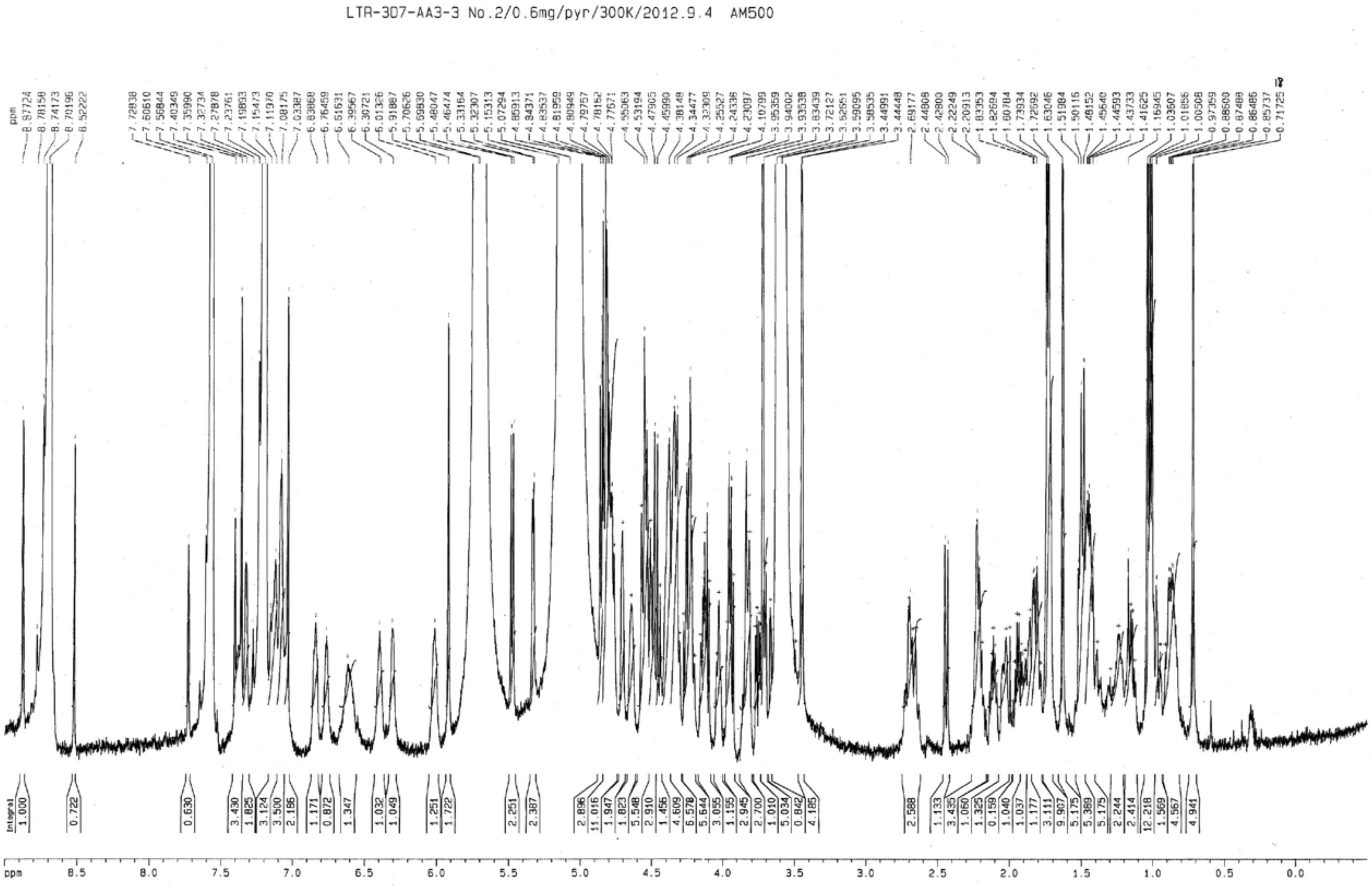

Figure S13. 1H-NMR spectrum of **4**.

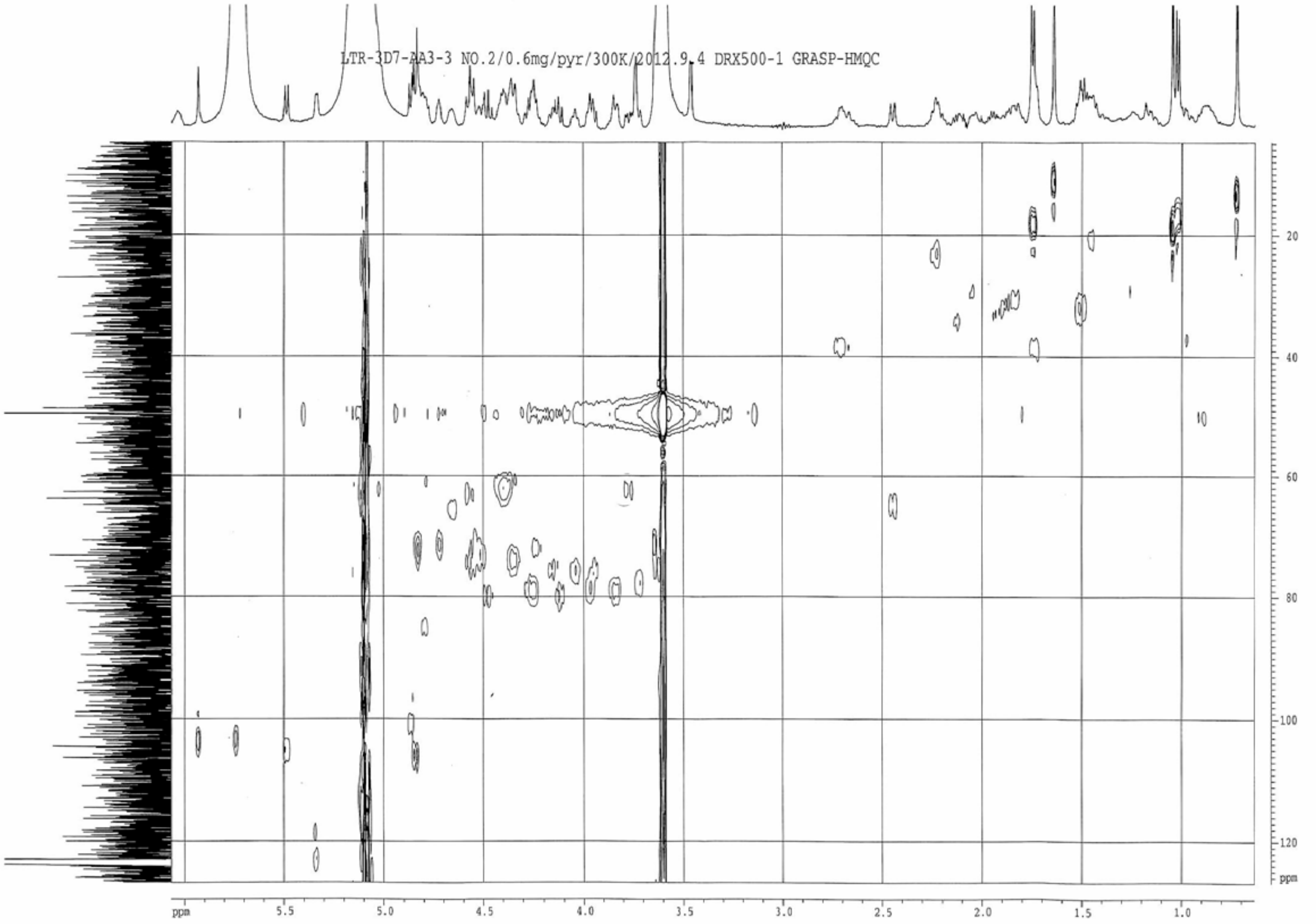

Figure S14. HMQC of **4**.

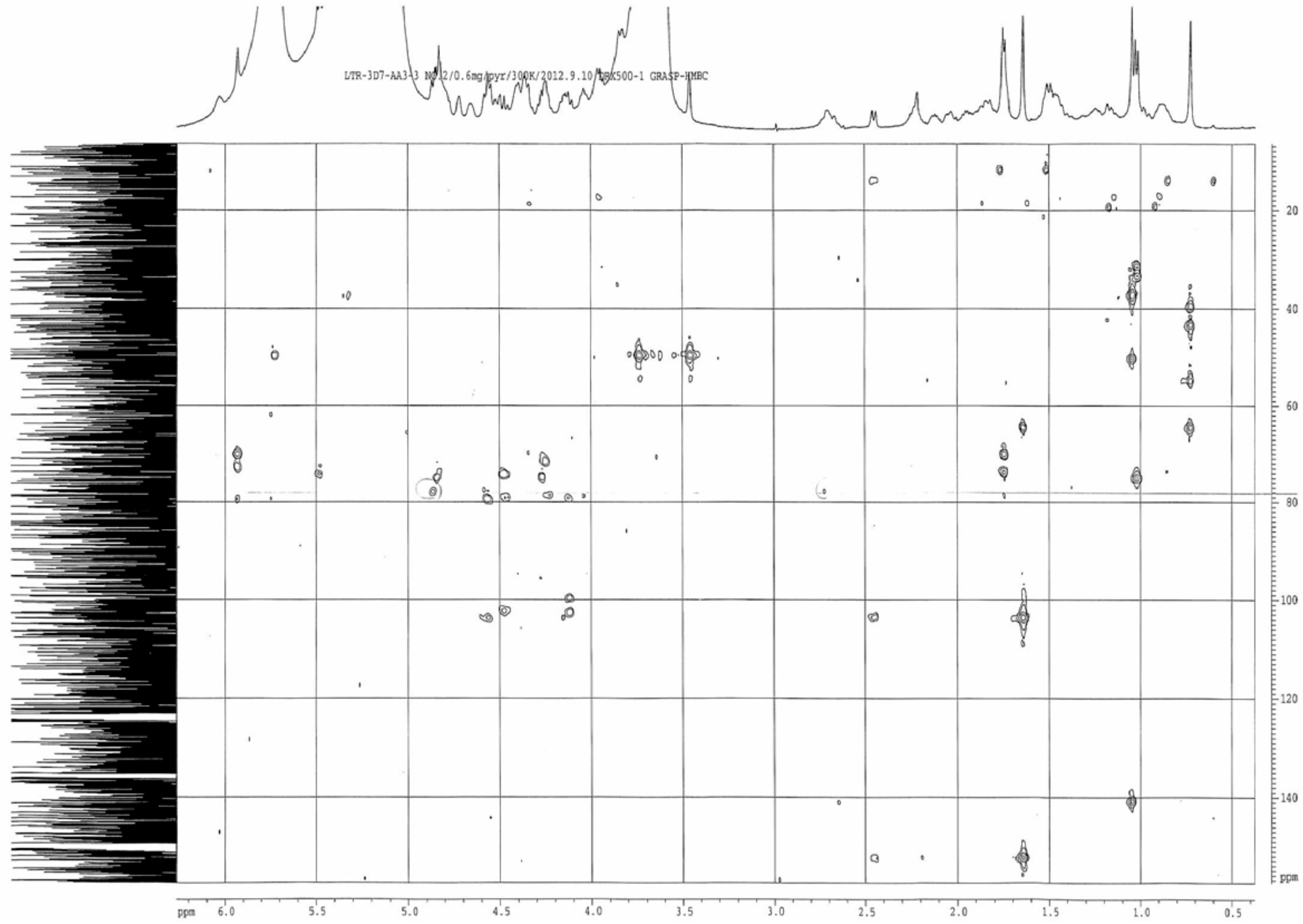

Figure S15. HMBC of **4**.

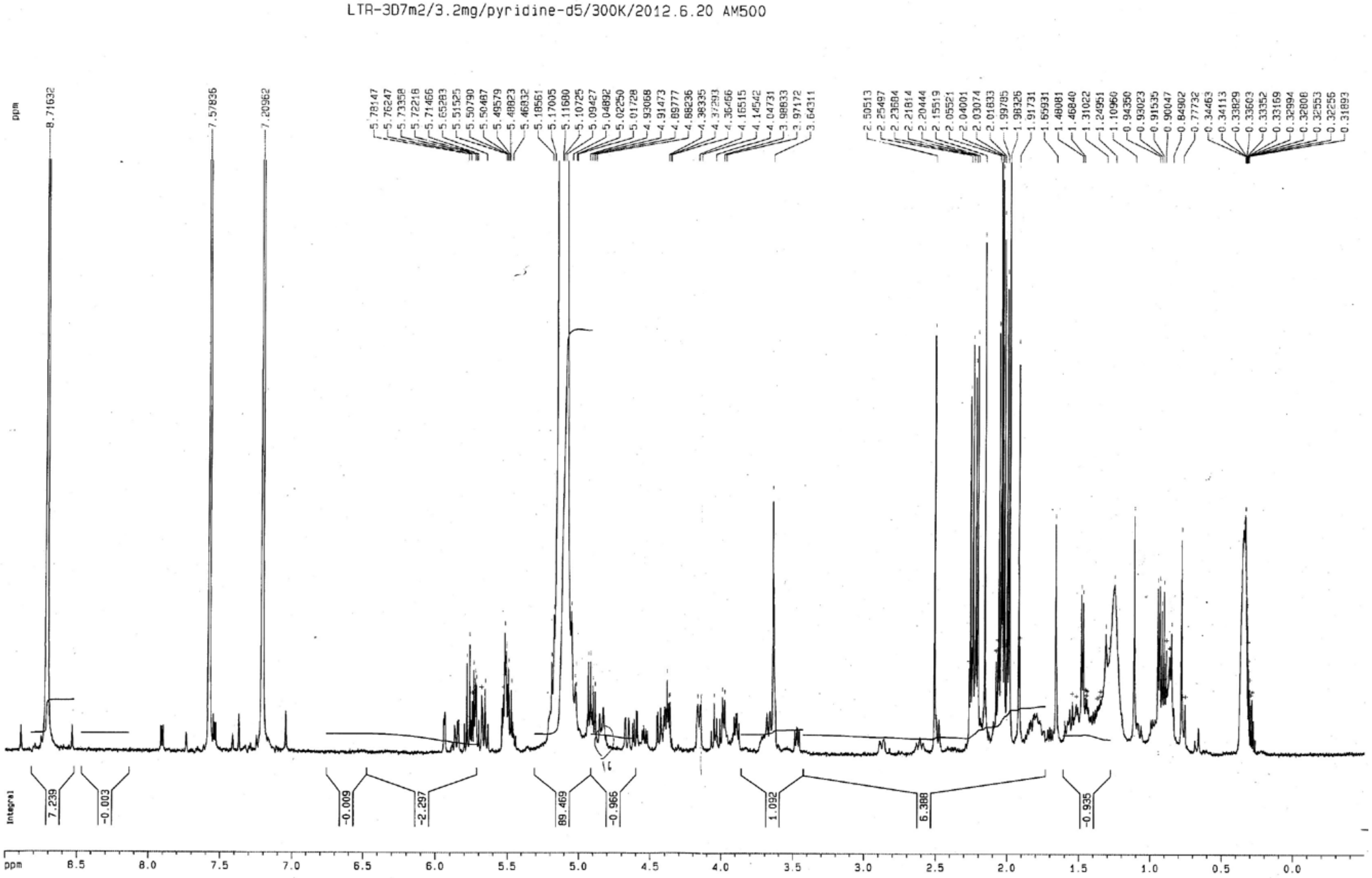

Figure S16. ^{1}H-NMR spectrum of **4a**.

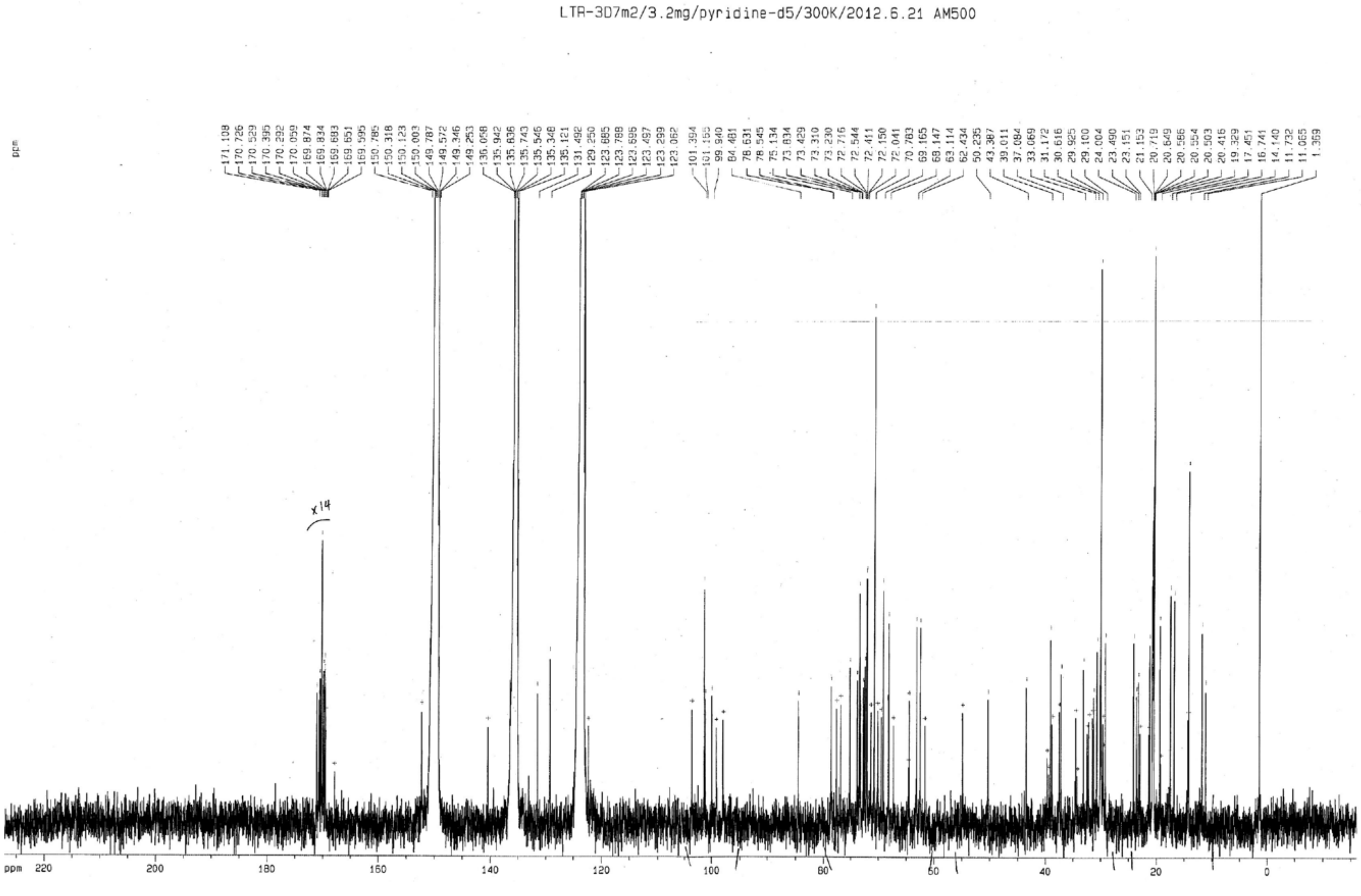

Figure S17. ^{13}C-NMR spectrum of **4a**.
